# Androgen and Oestrogen Affect the Expression of Long Non-Coding RNAs During Phallus Development in a Marsupial

**DOI:** 10.3390/ncrna5010003

**Published:** 2018-12-30

**Authors:** Yu Chen, Yoko Kuroki, Geoff Shaw, Andrew J. Pask, Hongshi Yu, Atsushi Toyoda, Asao Fujiyama, Marilyn B. Renfree

**Affiliations:** 1 School of BioSciences, The University of Melbourne 3010, VIC, Australia; y.chen61@student.unimelb.edu.au (Y.C.); g.shaw@unimelb.edu.au (G.S.); a.pask@unimelb.edu.au (A.J.P.); hongshiy@unimelb.edu.au (H.Y.); 2 RIKEN, Center for Integrative Medical Sciences, Yokohama, Kanagawa 230-0045, Japan; yoko-kuroki@amed.go.jp; 3 Advanced Genomics Center, National Institute of Genetics, Mishima, Shizuoka 411-8540, Japan; atoyoda@nig.ac.jp (A.T.); afujiyam@nig.ac.jp (A.F.)

**Keywords:** hypospadias, lncRNA, RNA-Seq, WGCNA, marsupial, androstanediol, phallus, androgen receptor, oestrogen receptor, castration

## Abstract

There is increasing evidence that long non-coding RNAs (lncRNAs) are important for normal reproductive development, yet very few lncRNAs have been identified in phalluses so far. Unlike eutherians, phallus development in the marsupial tammar wallaby occurs post-natally, enabling manipulation not possible in eutherians in which differentiation occurs in utero. We treated with sex steroids to determine the effects of androgen and oestrogen on lncRNA expression during phallus development. Hormonal manipulations altered the coding and non-coding gene expression profile of phalluses. We identified several predicted co-regulatory lncRNAs that appear to be co-expressed with the hormone-responsive candidate genes regulating urethral closure and phallus growth, namely *IGF1*, *AR* and *ESR1*. Interestingly, more than 50% of *AR*-associated coding genes and lncRNAs were also associated with *ESR1*. In addition, we identified and validated three novel co-regulatory and hormone-responsive lncRNAs: *lnc-BMP5, lnc-ZBTB16* and *lncRSPO4*. *Lnc-BMP5* was detected in the urethral epithelium of male phalluses and was downregulated by oestrogen in males. *Lnc-ZBTB16* was downregulated by oestrogen treatment in male phalluses at day 50 post-partum (pp). *LncRSPO4* was downregulated by adiol treatment in female phalluses but increased in male phalluses after castration. Thus, the expression pattern and hormone responsiveness of these lncRNAs suggests a physiological role in the development of the phallus.

## 1. Introduction

The 20,000 protein coding genes only account for about 1% of the human genome [1,2]. However, non-protein coding RNAs (ncRNAs) comprise at least three times more DNA than protein coding RNAs in the human genome [3,4,5,6]. Many ncRNAs participate in regulating genome function [7] and gene regulation [8] during all biological processes including sex determination and differentiation [9,10]. Long non-coding RNAs (lncRNAs) form a major class of non-coding transcripts [4,11] characterised by a transcript longer than 200 nucleotides with either no significant open reading frames (ORF) or with ORF shorter than 30–60 amino acids [12]. However, little is known about the interactions between protein coding genes and lncRNAs due to the low conservation of lncRNA sequences, lack of understanding of their regulation or what they regulate. Even well-identified mouse lncRNAs are generally not sequence conserved, with only 14% of lncRNAs that have a conserved orthologue in the human genome [13,14].

Long ncRNAs play an important role in gene regulation during appendage development, sexual differentiation and reproductive development (reviewed in [9,10,15,16]). A recent study suggests that anti-sense lncRNAs have an important role in regulating appendage development in mouse [16]. Many lncRNAs in the reproductive tract are androgen or oestrogen sensitive. For instance, *CTBP1-AS*, a lncRNA that promotes prostate cancer, is androgen responsive [17], whereas *NEAT1*, a lncRNA that also promotes prostate cancer, is oestrogen responsive [18]. The transcriptional regulation of lncRNAs can be either cis or trans (reviewed in [19]) so their targets are not always easily defined.

Unlike eutherian mammals, in which sexual differentiation takes place in utero, sexual differentiation occurs largely post-natally in marsupials. For the phallus, the initiation of genital tubercle (GT) outgrowth in the tammar begins 2 days before birth in both sexes [20]. From the day of birth to day 50 post-partum (pp), there are no morphological differences between male and female phalluses [21]. After day 50 pp, the distance between the urethral meatus and the anus becomes greater in males than in females [20,21,22] and the male GT elongates [23]. By day 150 pp, urethral closure is complete, with the urethral meatus in the glans penis. In contrast, the urethral groove does not fuse in females, as seen in other mammals [21,23].

Phallus development in the male tammar is androgen dependent [24] and can be affected by sex hormones [23,24,25]. Interestingly, the earliest sexually dimorphic differences occur at a time when circulating androgen levels are similar in both males and females [21,23,26,27,28], suggesting that hormonal-responsive pathways are activated before phallus differentiation. This hormone-sensitive window (also known as the androgen imprinting window or male programming window) [23,27,29] was the first of several now described in eutherian mammals and occurs between day 25 to 30 pp [25]. Altering androgen levels during this window of sensitivity in the tammar wallaby significantly altered gene expression in multiple signalling pathways, including SHH signalling, IGF1 signalling and AP-1 signalling, and also leads to hypospadias or sex-reversed phalluses at later stages [23,24,30]. The postnatal phallus development and androgen imprinting mechanism in the tammar makes it possible to administer androgen or oestrogen directly to pouch young without interference from the maternal physiology or placental transfer.

It is clear that altering sex steroids during the androgen imprinting window changes multiple signalling pathways and causes hypospadias in the tammar. Although several studies suggest that hormone-responsive lncRNAs regulate reproductive tract development by affecting coding genes [10,15,17,18], there are few studies on the regulation of hormone-responsive lncRNAs during phallus development. In this study, we used the marsupial tammar wallaby *Macropus eugenii* as a research model to study the co-expression network between hormone-responsive coding genes and lncRNAs during phallus development. We investigated the regulation of multiple hormone-responsive lncRNAs that are co-expressed with coding genes in the developing tammar phallus. We treated female tammars with androgen and males with oestrogen or castration during the androgen imprinting window of sensitivity to identify hormone-responsive lncRNAs. We identified novel putative co-regulatory hormone-responsive lncRNAs of three genes regulating phallus development: insulin growth factor (*IGF1*), androgen receptor (*AR*) and estrogen receptor alpha (*ESR1*). *IGF1*, one of many hormone-responsive coding genes [31], may induce urethral closure by activating cell proliferation in the urorectal septum of male tammar phalluses and enhance growth [31]. In the mouse, the balance between AR and ESR1 activity is important to maintain normal penile development [32]. We used co-expression analysis to identify novel co-regulatory hormone-responsive lncRNAs during phallus development. Our data demonstrate that hormonal exposures have a significant impact on the network of coding and lncRNAs during phallus development, which extends our understanding on the aetiology of abnormal phallus development, including hypospadias.

## 2. Results

### 2.1. Principal Component Analysis

**Figure 1:**
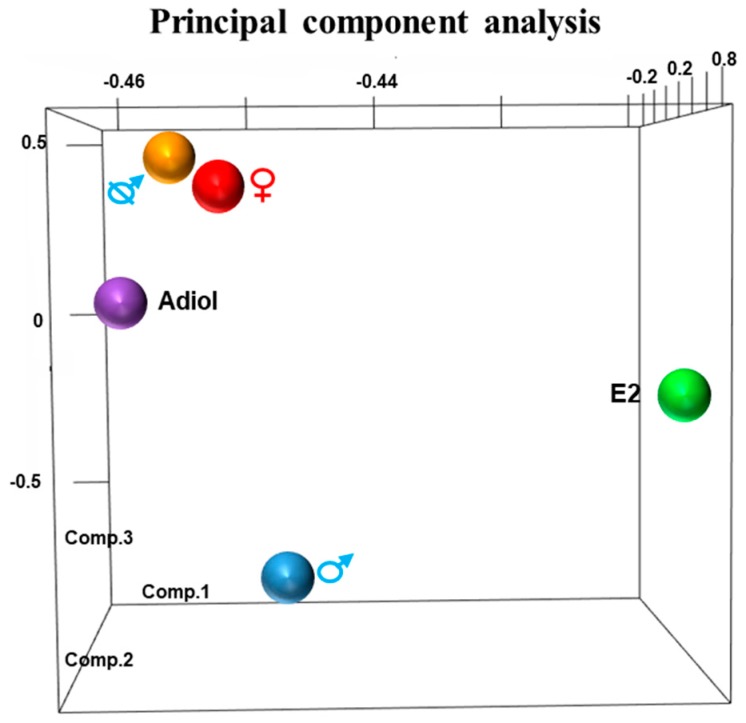
Principal component analysis (PCA) demonstrates the variance of expression profile between treated phalluses and normal phalluses. Principal component 1–3 represent for 94.3%, 4.5% and 0.73% of the variance. Adiol and oestrogen treatment altered the gene expression pattern away from that in normal day 50 post-partum (pp) female and male phalluses, respectively. The gene expression pattern in oestrogen treatment group was different from that in adiol treatment group. Castration has mostly reversed the gene expression pattern in the phallus from males to females. E2: oestrogen.

The 3-dimensional (3-D) principal component analysis (PCA) of RNA-Seq data separated the response of each treatment from the control groups (Figure 1). There was a sexually dimorphic expression pattern between day 50 pp male and female phalluses. In males treated with oestrogen, the phallus gene expression pattern diverged from that of normal day 50 pp males. Similarly, in females treated with adiol, the phallus expression pattern differed from normal day 50 pp female phalluses. Castration resulted in a pattern more like females than males. About 14% of these differentially expressed genes presented above were predicted lncRNAs (Table S1, see Supplementary Materials).

### 2.2. Predicted Hormone-Responsive long non-coding RNAs

**Figure 2:**
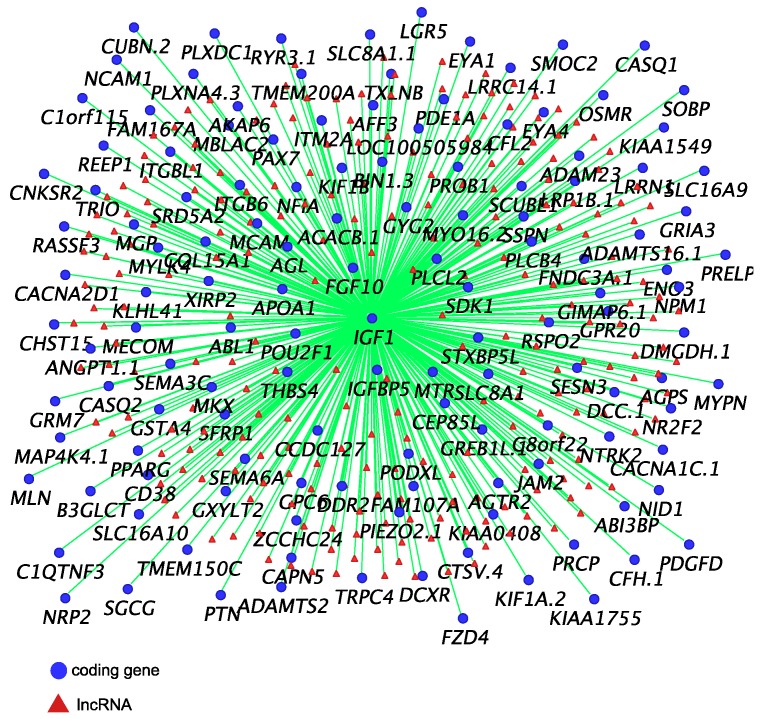
*IGF1* co-expressed coding genes and predicted co-regulatory long non-coding RNAs (lncRNAs) (R ≥ 0.8).

**Figure 3:**
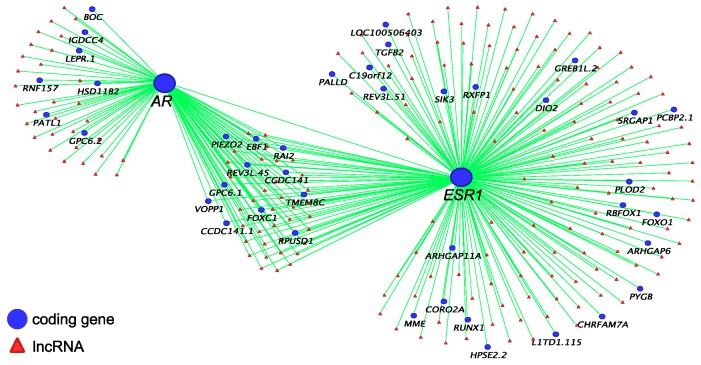
*AR* and *ESR1* co-expressed coding genes and predicted co-regulatory lncRNAs (R ≥ 0.7).

Co-expression networks of *IGF1* (Figure 2), *AR* and *ESR1* (Figure 3) were built to identify their potential co-regulatory lncRNAs. *IGF1* was the hub gene in one of 16 modules produced by co-expression analysis. The expression correlation between identified lncRNAs and *IGF1* was >0.8, and between identified lncRNAs and *AR* and *ESR1* was >0.7. More than 50% of *AR*-associated coding genes and lncRNAs were also associated with *ESR1* (Figure 3). However, none of these lncRNAs were located within 100 kilobases (kb) up-or down-stream of *IGF1, AR* and *ESR1*.

**Figure 4:**
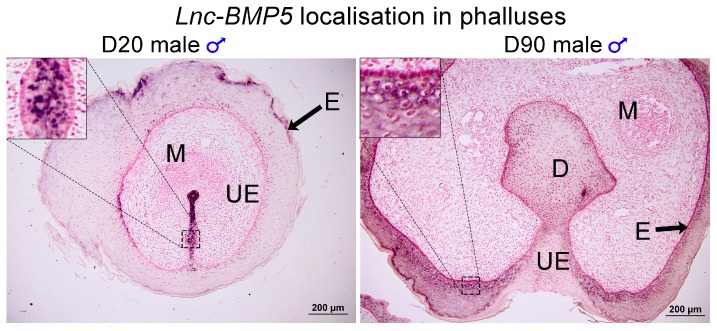
*Lnc-BMP5* mRNA localisation in day 20 pp and day 90 pp male phalluses. *Lnc-BMP5* was detected in the nucleus of urethral epithelial cells of male phalluses at day 20 pp and in male phallus epithelium at 90 pp. D: diverticulum, E: epithelium, M: mesenchyme, UE: urethral epithelium. Scale bar: 200 µm.

**Figure 5:**
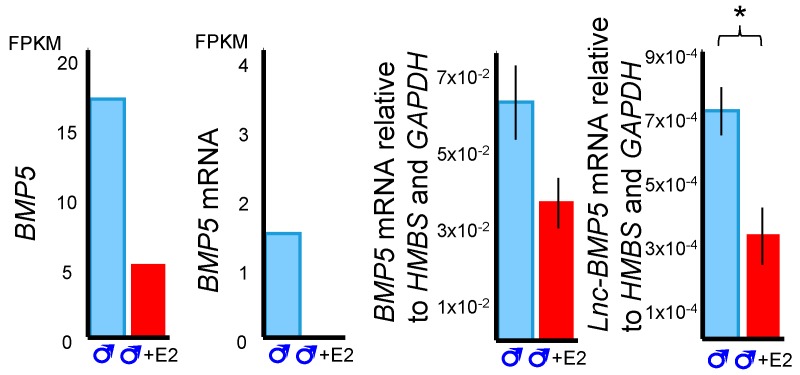
*BMP5* and *lnc-BMP5* expression from RNA-Seq (left) and validated in quantitative PCR (qPCR) (right). *BMP5* and *lnc-BMP5* was downregulated by oestrogen in males. E2: oestrogen. FPKM: Fragments Per Kilobase of transcript per Million mapped reads. *: *p* < 0.05, ***: *p* < 0.001. Error bar: SEM.

**Figure 6:**
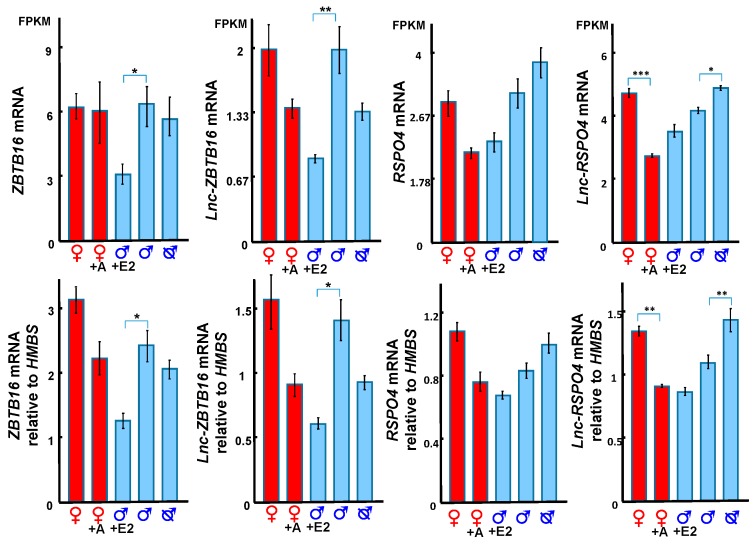
*ZBTB16*, *lnc-ZBTB16*, *RSPO4* and *lnc-RSPO4* transcript expression from RNA-Seq (top) and gene expression validated by qPCR (bottom). *ZBTB16* and *lnc-ZBTB16* were both downregulated by oestrogen in males. *Lnc-RSPO4* was downregulated in females by adiol treatment, but upregulated in males after castration. A: androgen, E2: oestrogen. FPKM: Fragments Per Kilobase of transcript per Million mapped reads. *: *p* < 0.05, **: *p* < 0.005, ***: *p* < 0.001. Error bar: SEM.

Three predicted co-regulatory lncRNAs, *lnc-BMP5, lnc-ZNTB16* and *lnc-RSPO4*, were identified by mapping co-expressed coding genes and lncRNAs in the tammar genome. The expression correlation between *lnc-BMP5, lnc-ZNTB16* and *lnc-RSPO4* and their neighbouring coding genes, *BMP5*, *ZBTB16*, and *RSPO4*, respectively, was >0.7 and the correlation coefficient was significant between those coding genes and lncRNAs. *Lnc-BMP5* was localized around 1 kb downstream of Bone Morphogenetic Protein 5 *(BMP5)* (Figure S1). It was detected in the urethral epithelium of male phalluses at day 20 pp and in the epithelium of the male phallus at day 90 pp, suggesting it may be involved in phallus development (Figure 4). *BMP5* and *lnc-BMP5* were downregulated by oestrogen in males (Figure 5). *Lnc-ZBTB16* was within 1 kb downstream of zinc finger and BTB domain containing 16 *(ZBTB16)* (Figure S1). Both *lnc-ZBTB16* and *ZBTB16* had similar transcriptome and quantitative PCR (qPCR) expression patterns (Figure 6).

*Lnc-ZBTB16* and *ZBTB16* were significantly downregulated after oestrogen treatment (Figure 6). *Lnc-RSPO4* was located within 1 kb upstream of R-Spondin 4 (*RSPO4)* (Figure S1). *Lnc-RSPO4* and *RSPO4* had similar qPCR and transcriptome expression patterns (Figure 6). *Lnc-RSPO4* was significantly down-regulated after adiol treatment (Figure 6). Although *RSPO4* expression was not as significantly changed as that of *lnc-RSPO4*, the expression correlation between *RSPO4* and *lnc-RSPO4* was significant.

## 3. Discussion

This study in the tammar suggests that phallus differentiation is under the control of complex network interactions between hormone-responsive coding genes and lncRNAs. Multiple predicted lncRNAs of important phallus regulating genes, including *IGF1, AR* and *ESR1,* were identified from co-expression analysis. We predicted and validated 3 novel co-regulatory lncRNAs (*lnc-BMP5, lnc-ZBTB16* and *lnc-RSPO4*). Those lncRNAs were all hormone-responsive and were located near their co-expressed and hormone-responsive coding genes in the tammar genome. 

### 3.1. A Global View of Differentially Expressed Genes and Phallus Phenotype

Three-dimensional PCA grouped the transcriptomic responses to treatments by phenotype showing a similar relationship to the morphological changes of phalluses as measured by qPCR in our previous study in the tammar [30]. Both adiol treatment in females and oestrogen treatment in males induce urethral closure in a micro-penis [23,30]. Similarly, in the 3-D PCA results, both adiol treatment of females and oestrogen treatment of males had a gene expression pattern distinct from that of normal female and male phalluses, respectively. Although both treatments resulted in a micro-penis with a closed urethra [30], the 3-D PCA highlighted differences between the adiol and oestrogen treatment groups. It is possible that the presence of their intact testes in the oestrogen-treated males counteracted some of the oestrogenic effects.

### 3.2. Co-Regulatory lncRNAs of IGF1, AR and ESR1

*IGF1* plays an important role in regulating phallus development in humans [33,34] and its sexually dimorphic expression suggests it may be equally important in the tammar phallus [31]. The balance between local expression of *IGF1* and insulin growth factor binding proteins (*IGFBPs)* is required to maintain the IGF1 signalling pathway during normal tissue growth [31,35,36]. Our co-expression analysis showed that *IGF1* was highly correlated (R > 0.8) with *IGFBP5* and Fibroblast Growth Factor *(FGF10)*, another important growth factor in regulating phallus development in mice [37,38,39,40] and implicated in the tammar [31]. Similarly, a large group of predicted lncRNAs were also highly correlated (R > 0.8) with *IGF1* expression. However, little is known about the functional role of those lncRNAs during reproductive tract development. *AR* was correlated with *ESR1* and more than 50% of *AR*-correlated coding genes and lncRNAs were also correlated with *ESR1*. These data further confirm a potential interaction between *AR* and *ESR1* networks during phallus development, as seen in mice [32]. Interestingly, none of those lncRNAs were located within 100 kb up-or down-stream of *IGF1, AR* and *ESR1*, suggesting that those lncRNAs may have other gene targets.

### 3.3. BMP5 and Lnc-BMP5 in Phallus Development

Bone morphogenetic proteins (BMPs), especially Bmp7, play an important role in regulating urethra formation in mice [41,42,43,44,45]. Loss of *Bmp7* signalling causes hypospadias [41,43]. In contrast, some BMPs, such as Bmp4, suppress urethral outgrowth, increase apoptosis and inhibit cell proliferation of the genital tubercle mesenchyme in mice [45]. Several *BMPs* are hormone-responsive during mouse phallus development [32]. In male mice treated with flutamide, an androgen receptor inhibitor, expression of *Bmp5* decreases but *Bmp2* expression increases [32]. Male mice exposed to oestradiol benzoate, a synthetic oestrogen, upregulated *Bmp8b* [32]. Interestingly, *BMP5*, and its neighbouring *lnc-BMP5* were downregulated by oestrogen treatment during tammar phallus sexual differentiation. In addition, *lnc-BMP5* was localised in the urethral epithelium of tammar phalluses. Our data suggested that *BMP5* and *lnc-BMP5* were susceptible to oestrogen treatment, suggesting these might be another pair of genes that regulate phallus development, similar to the other *Bmps* mentioned above.

### 3.4. Hormonal Regulation of ZBTB16 and its Correlated lncRNA

We identified a new lncRNA, *lnc-ZBTB16* within 1kb upstream of ZBTB16, which had the highest correlation with *ZBTB16* expression. *ZBTB16* gene encodes a zinc finger transcription factor and the missense mutation of *ZBTB16* induces micro-penis in boys [46,47], indicating an important role for this gene during genital development. With co-expression analysis, we showed that *ZBTB16* and *lnc-ZBTB16* were both downregulated by oestrogen, suggesting a functional relationship between the two. Interestingly, oestrogen treatment in the tammar from day 20–40 induces a micro-penis by day 150 pp [30], similar to boys with the missense mutation of *ZBTB16* [46,47]. We suggest that *ZBTB16* and its co-expressed *lnc-ZBTB16* might be the primary target of oestrogen that induces micropenis. 

### 3.5. RSPO4 and lnc-RSPO4 May Regulate Phallus Development Through Activating WNT5A

RSPO4, a ligand of leucine rich repeat containing G protein-coupled (LGR) 4–6 receptors, potentiates the Wingless-Type MMTV Integration Site Family Member (WNT)/β-catenin signalling pathway [48,49,50,51]. The WNT/β-catenin signalling pathway interacts with androgens and hedgehog signalling to regulate normal phallus development in mammals [30,32,52,53,54,55,56,57]. In the tammar, *RSPO4* and *lnc-RSPO4* were downregulated in adiol treated female phalluses at day 50 pp, but upregulated in phalluses of earlier castrated males at day 50 pp. The expression pattern of *RSPO4* and *lnc-RSPO4* was similar to that of *WNT5A* and *SHH* [25,30]. *RSPO4* and *lnc-RSPO4* may also be involved in the interaction between androgen, *SHH* and *WNT5A* driving masculinization of the phallus, as occurs in knockout mice [52,53]. Our data demonstrate that there is a complex network of hormonally-regulated coding and lncRNA regulatory transcripts during phallus development, extending our understanding of the aetiology of abnormal phallus development, including hypospadias and potential effects of environmental steroids on the reproductive system.

This study confirms that exogenous hormonal manipulation altered the expression pattern of genes, and highlighted the importance of both androgen and oestrogen in phallus development. We identified multiple co-regulatory lncRNAs with their associated coding genes, such as *IGF1, AR* and *ESR1*. There appears to be an interaction between AR and ERα signalling during phallus development in the tammar, as seen in mice. Furthermore, we validated three novel co-regulatory hormonal responsive lncRNAs co-expressed with *BMP5, ZBTB16* and *RSPO4* that may also regulate phallus differentiation, as seen in mice. The results demonstrate that tammar phallus differentiation is under a complex regulation network of protein-coding genes and their co-expressed lncRNAs, all of which are susceptible to exogenous sex steroids.

## 4. Materials & Methods

### 4.1. Animals

Tammar wallabies (*Macropus eugenii*) of Kangaroo Island (South Australia) origin were kept in our breeding colony in Melbourne. The sex of the young was identified by the presence of scrotal or mammary primordia [58]. The age of the pouch young was estimated from measurements of head length and weight from published growth curves [59]. Phalluses were collected from pouch young tammar wallaby after anaesthesia with Zoletil 100 (Tiletamine HCl 50 mg/mL, Zolazepam HCl 50 mg/mL, Virbac, Cat# 1p6080-3, 1 mL/kg,) and killing by an overdose of sodium pentobarbitone (60 mg/mL, to effect). Samples were snap-frozen and stored at −80 °C for RNA-Seq analysis or fixed in 4% (*w*/*v*) paraformaldehyde, embedded in paraffin, and cut into 7 µm sections for histological analysis and section in situ hybridization. All experimental procedures complied with the Australian National Health and Medical Research Council (2013) guidelines and approved by the University of Melbourne Animal Experimentation Ethics Committees. Phalluses for section in situ hybridisation (*n* = 3) were collected as above from day 20 pp and day 90 pp males. Male pouch young were castrated (*n* = 5) as previously described [23,25,60] or treated with oestradiol benzoate (2.5 mg/kg/day, dissolved in triolein oil, Cat#50-50-0, Sigma, St. Louis, MO, USA) orally from day 20 pp to day 40 pp (*n* = 5) as previously described [61]. Female pouch young (*n* = 5) were injected with 5α-androstane-3α,17β-diol (adiol, 10 mg/kg/week, dissolved in triolein oil, Cat#A1170-000, Steraloids, Newport, USA) from day 20 pp to day 40 pp as previously described [23]. The treated pouch young and control phalluses were collected at day 50 pp (*n* = 5) for RNA-Seq analysis and qPCR (*n* = 5).

### 4.2. Transcriptome Analysis

Two RNA-seq data-sets were used: one, standard 100bpx2 RNA-seq (no replicates but a single pool of 5 samples for each tissue) and the other, strand-specific (5 replicates of each tissue). RNA samples were further tested using Bioanalyzer (Cat#G2939BA, Agilent, Santa Clara, CA, USA). Appropriate amounts of RNA were subjected to multiplex indexed-RNA-Seq analyses using TruSeq RNA Sample prep Kit (Cat#15008136A, Illumina, San Diego, CA, USA) or TruSeq Stranded Total RNA Prep Kit (Cat#20020596, Illumina, San Diego, CA, USA), and HiSeq2000 analyser (Cat#15011190, Illumina, San Diego, CA, USA). Roughly 130–150 × 10^6^ reads (100 bp each from the both ends) were obtained for each index-tag. Reads were obtained from each sample after quality control (Q.C.) > 30 filtering. The average quality score was ~39. The RNA-Seq data were assembled with Trimmomatic [62] and Cufflinks-Cuffdiff [63] pipeline and analysed with CummeRbund R package [64]. The average of mapping rate from all samples was above 80% and the quality was good for differential expression analysis. The original (Renfree et al., 2011) and the updated tammar wallaby genome 3.0 [65] were used as a guide for transcriptome assembly. The annotation was obtained by blasting against the UNIPROT protein database [1]. Differentially expressed genes were extracted with CummeRbund R package and basic R scripts [66]. 3-D PCA analysis used the R package rgl [67]. Principal components 1–3 represent 94.3%, 4.5% and 0.73% of the variance. Long ncRNAs were predicted by using protein potential calculator [68]. *BMP5* and *lnc-BMP5* were extracted from the standard 100bpx2 RNA-seq data-set (no replicate single pool), whereas *RSPO4, lncRSPO4, ZBTB16* and *lnc-ZBTB16* were extracted from the strand-specific RNA-seq data-set (5 replicates).

### 4.3. Weighted Gene Co-Expression Network Analysis

Co-expression analysis between protein coding genes and lncRNAs were conducted by Weighted Gene Co-Expression Network Analysis (WGCNA), R package [69]. RNA-Seq data was normalised (log-transformed) before entering WGCNA pipeline [70]. WGCNA produced 16 different modules named with different colours. The co-expression figure was produced by using VisANT [71]. The target genes of the co-expression network were selected based on their correlation coefficient in each colour-coded module and their importance in phallus development from all relevant publications cited in this paper. *IGF1* was analysed in a different module (Figure 2) from *AR* and ESR1 (Figure 3). The co-regulatory lncRNAs were selected based on their correlation coefficient (R ≥ 0.7) with *IGF1, AR* and *ESR1*, if they were not located on the same scaffold of those coding genes. The co-regulatory and hormone-responsive lncRNAs were selected by firstly filtering with differentially expressed lncRNAs extracted from RNA-Seq into each co-expressed module (Figure S2), then selected based on their correlation coefficient (R ≥ 0.7) with the coding genes and whether they are located within 100 kb upstream or downstream of respective coding genes.

### 4.4. RNA Extraction and cDNA Preparation

RNA was extracted from developing phalluses with the RNeasy Mini kit (Cat# 74804, QIAgen, Hilden, Germany) following on the manufacturer’s recommendations. The RNA was calculated with a Qubit 2.0 fluorometer (Cat#Q32866, ThermoFisher Scientific, Waltham, MA, USA). All RNA samples were treated with DNA-free enzyme (Cat#1906, ThermoFisher Scientific) to prevent genomic DNA contamination. 200 ng of total RNA was used for complementary DNA (cDNA) template synthesis by using transcription high fidelity cDNA Synthesis kit (Cat# 05081963001, Invitrogen, Waltham, MA, USA).

### 4.5. Quantitative PCR

FastStart Universal SYBR mix (Cat#04913914001, Roche, Basel, Switzerland) was used to detect gene expression level. PCR primers (Table S2) were designed with program of Primer 3 online (http://primer3.ut.ee/). The estimated efficiency of each set of primers was within 95%~105%. The amplification conditions for qPCR were: 1 cycle of 95 °C for 10 min; 45 cycles of 95 °C for 30 s, 58 °C for 30 s, 72 °C for 1 min; and 1 cycle of 72 °C for 7 min. All genes were run with 5 replicates. Two house-keeping genes (*HMBS* and *GAPDH*) were used to normalise the expression level. The method that we used to calculate the expression level was described in the MIQE guidelines [72,73]).

### 4.6. Section in situ Hybridization

Phalluses for section in situ hybridization were collected from day 20 pp and day 90 pp pouch young (*n* = 3 each stage). The paraffin embedded phalluses were sectioned at 7 µm. Primers were designed with online program of Primer 3 online (http://primer3.ut.ee/) (lnc-BMP5: forward: 5′-GCCAGTTTCCAGACTTTGTGA-3′, reverse: 5′-GGAGCTTTGACTTTGTTTTCTTC-3′). The probes for in situ hybridization (ISH) were labelled with DIG RNA labelling Mix (Roche, Cat#11277073910) and generated with T7/SP6 polymerase synthase kit (Promega, Cat #P1460). The sections were pre-hybridized for 2 h at 42 °C and hybridized for 16–18 h at 42 °C, sections washed and incubated with Anti-Digoxigenin-AP (1:300 dilution, Roche, Cat #11093274910) for 16–18 h at 4 °C and colour developed with NBT/BCIP (1:50 dilution, Roche, Cat #11681451001). The sections were counter-stained with Nuclear Fast Red solution (Cat #N3020 Sigma Aldrich, St. Louis, MO, USA). Negative controls were incubated with sense probe (Figure S3).

### 4.7. Statistics

Three replicates were used for section in situ hybridisation. Five replicates were used for qPCR and RNA-Seq analysis. FPKM (fragments per kilobase of exon model per million reads mapped) is a normalised estimation of gene expression based on RNA-Seq data. FPKM are calculated from the number of reads that mapped to each particular gene sequence taking into account the gene length and the sequencing depth. Student’s *t*-test was used to calculate the significance (*p* < 0.05) of normalized RNA-Seq data and qPCR data. The correlation of *IGF1* in black module is 0.944. The correlation of *AR* and *ESR1* in green module are 0.655 and 0.766. The *p*-values of the correlation coefficient between coding genes and lncRNAs are lower than 0.0001 in their respective module. All error bars represent standard error of the mean (SEM).

## Supplementary Materials

The following are available online at http://www.mdpi.com/2311-553X/5/1/3/s1. Figure S1: Genomic location of *lnc-BMP5, lnc-ZBTB16* and *lnc-RSPO4* in the tammar wallaby. Figure S2. Differentially expressed lncRNAs co-expression network (R ≥ 0.7). Figure S3. Negative control of *lnc-BMP5* in situ hybridisation result in day 20 pp and day 90 pp male phalluses. CC: corpus cavernosa, E: epithelium, M: mesenchyme, UE: urethral epithelium. Scale bar: 500 μm. Table S1: List of gene expression in RNA-Seq data. Table S2: List of primers used for qPCR.
