# Supplementary material for: Androgen and Oestrogen Affect the Expression of Long Non-Coding RNAs During Phallus Development in a Marsupial
Source: Noncoding RNA. 2018 Dec 30;5(1):3. doi: 10.3390/ncrna5010003 (PMC6468475; doi:10.3390/ncrna5010003)
Supplement: Supplementary file 1 [file ncrna-05-00003-s001.zip › Supplementary Table S2.pdf]

Table 1: List of primers used for qPCR

Gene	Primer sequence
BMP5 forward	CCGGATATACAAAGACCGCAGCAA
BMP5 reverse	TTCTCGCGTCTAACAGCAACAGG
lnc-BMP5 forward	AAGACCTGCGTGAGACAGTAGAG
lnc-BMP5 reverse	TGTGAGCCTAGCACTCCATAAACA
ZBTB16 forward	CTGCACTATGGACGGGAAGAGA
ZBTB16 reverse	GGGAAGGACAGAGTAGATGCCC
lnc-ZBTB16 forward	TGAAGGAAAGAGGGAGAGGAGAG
lnc-ZBTB16 reverse	GGGCTGTGAGGGTAAAGAGAGG
RSPO4 forward	TCCTGCTCACACTGCTGCTATTC
RSPO4 reverse	GCCCGACTCCCACTTGTTTCTT
lnc-RSPO4 forward	TCTCCTGCTCCTTTGTCCCGAA
lnc-RSPO4 reverse	ATCCCTCTCTGCTCCCTATGCC
