# Supplementary figures and images for: Androgen and Oestrogen Affect the Expression of Long Non-Coding RNAs During Phallus Development in a Marsupial
Source: Noncoding RNA. 2018 Dec 30;5(1):3. doi: 10.3390/ncrna5010003 (PMC6468475; doi:10.3390/ncrna5010003)

***BMP5* neighbouring *lncRNA***

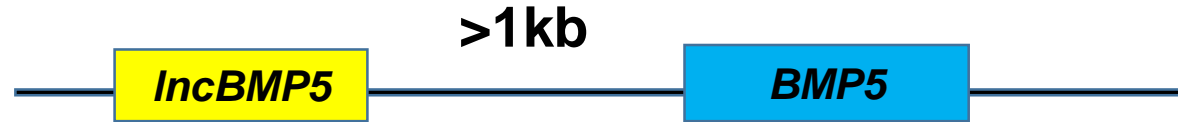

***ZBTB16* neighbouring *lncRNA***

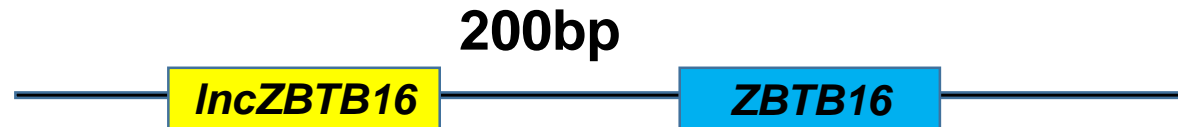

***RSPO4* neighbouring *lncRNA***

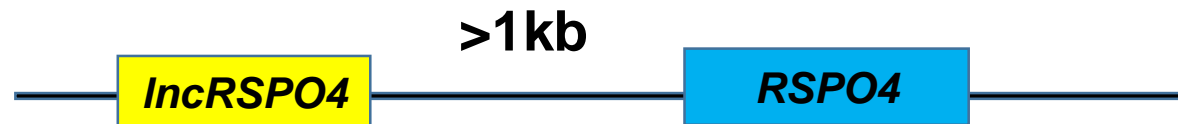

Supplement: Supplementary file 1 [file ncrna-05-00003-s001.zip › Supplementary Figure S1.pdf]

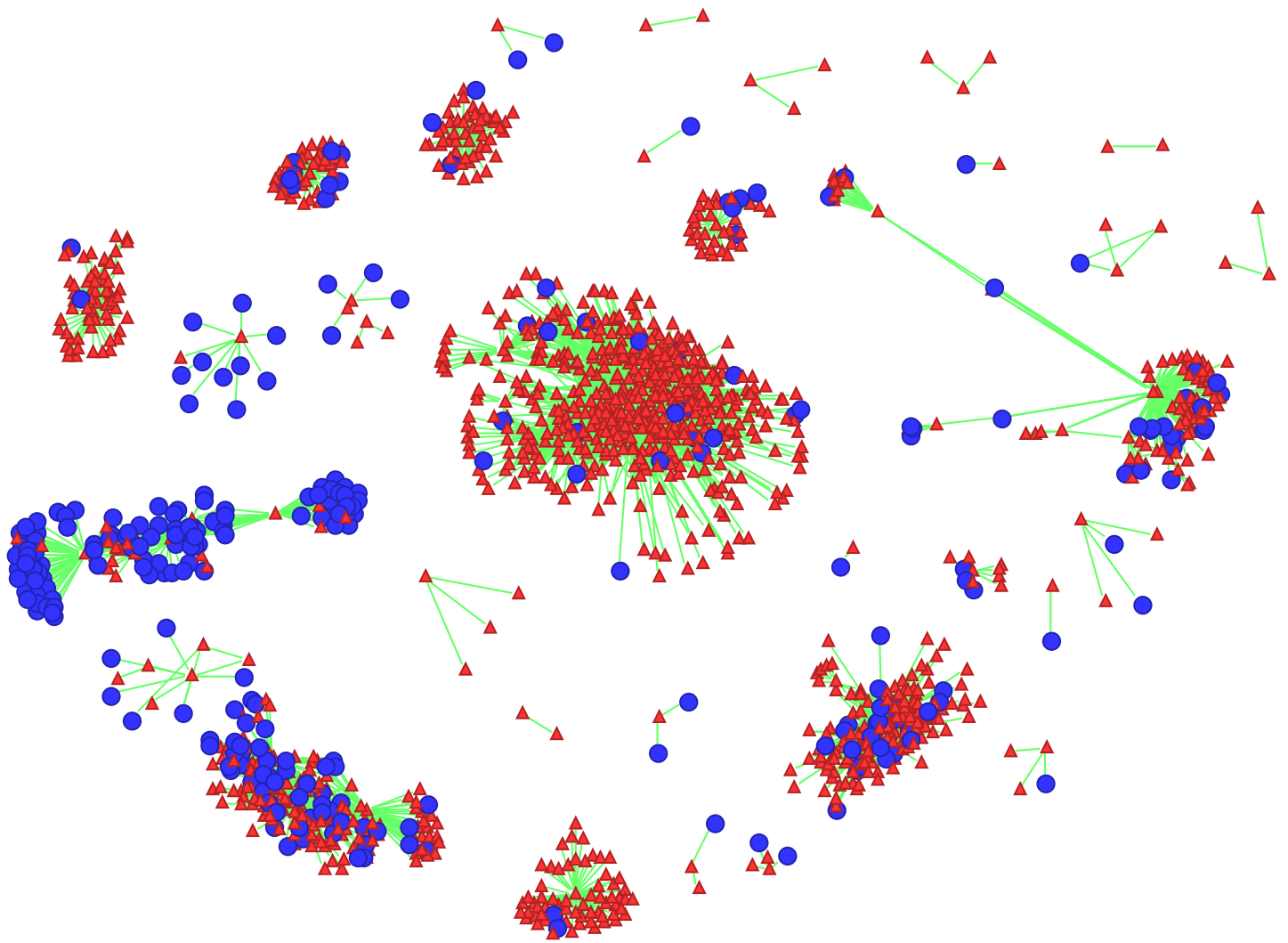

● coding gene  
▲ lncRNA

Supplement: Supplementary file 1 [file ncrna-05-00003-s001.zip › Supplementary Figure S2.pdf]

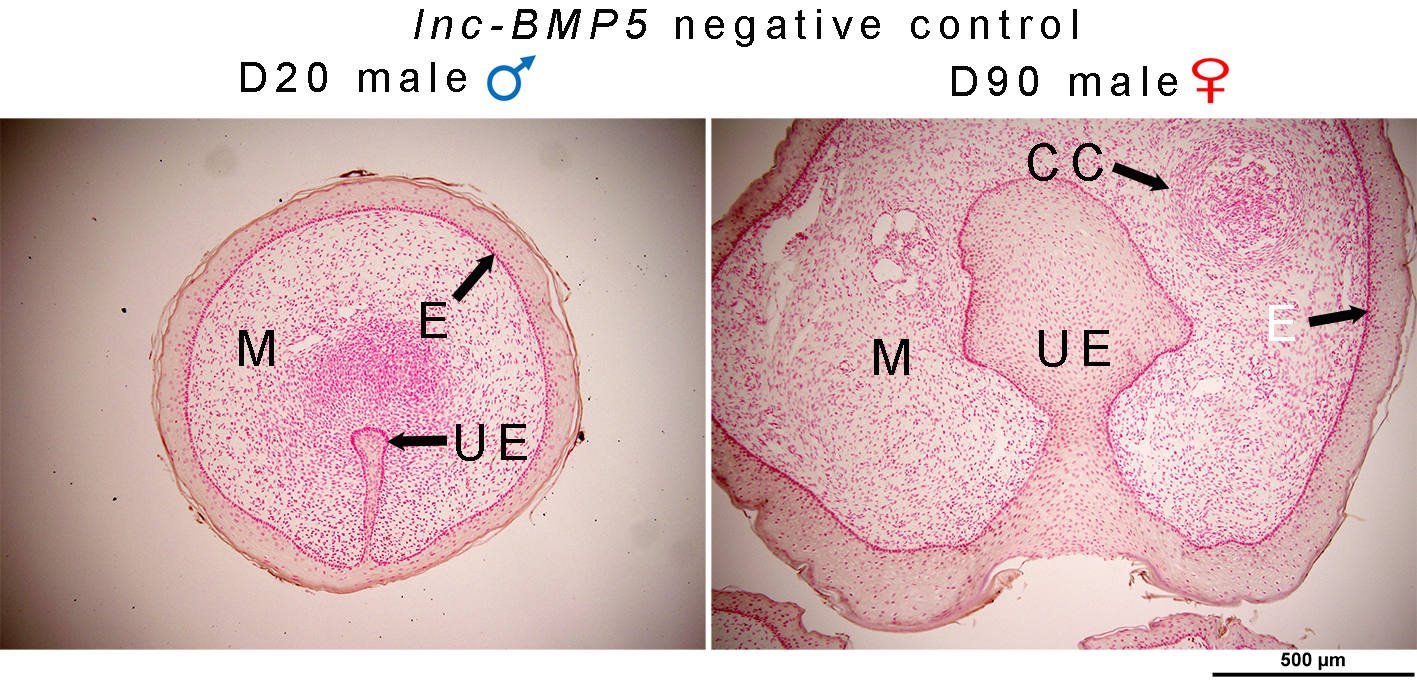

Supplement: Supplementary file 1 [file ncrna-05-00003-s001.zip › Supplementary Figure S3.tif]
